# Highlight report: Activating tumor-specific T-cells for breast cancer therapy

**DOI:** 10.17179/excli2018-2031

**Published:** 2018-12-27

**Authors:** Mikheil Gogiashvili

**Affiliations:** 1 Leibniz-Institut für Analytische Wissenschaften - ISAS - e. V., Bunsen-Kirchhoff-Str. 11, 44139 Dortmund

## ⁯⁯⁯⁯⁯

In breast cancer, the presence of tumor-infiltrating lymphocytes (TILs) is reported to confer a survival advantage (Schmidt et al., 2018[16]; Heimes et al., 2017[5][6]). Usually, increased infiltration with T- or B-cells is associated with better prognosis (Schmidt et al., 2008[14]; Chen et al., 2012[2]; Godoy et al., 2014[4]), and it has also been associated with better response to neoadjuvant chemotherapy (Schmidt et al., 2012[15]; Sota et al., 2014[18]). Factors related with an immunosuppressive microenvironment, such as elevated adenosine levels and high expression of the ectonucleotidase CD73 (Leone and Emens, 2018[10]), have contrarily been shown to be associated with worse prognosis in breast cancer (Jiang et al., 2018[7]). It has also been reported that tumors can suppress the anti-cancer immune response by the regulation of lactic acid production, and maintenance of a relatively low pH in the tumor microenvironment (Choi et al., 2013[3]), further stressing the importance of immune regulatory factors in cancer.

Recently, immune checkpoint inhibitors, targeting CTLA-4 and the PD-1/PD-L1 axis have been shown to generate long-lasting responses in several cancer types (Pennock and Chow, 2015[13]). For instance, CTLA-4 is known to abrogate the activated T-cell response, and antagonizing this mechanism represents an emerging anti-cancer strategy (Sharma and Allison, 2015[17]; Melero et al., 2014[12]). Many breast cancers exhibit relatively low T-cell infiltration together with a low neoantigen burden, as well as inadequate T-cell priming and expansion; thus they have been referred to as “immunologically cold" (Vonderheide et al., 2017[19]). Therefore, activation and expansion of tumor-specific T-cells represents a major focus in cancer research (Xu et al., 2013[20]; Sharma and Allison, 2015[17]; Melero et al., 2014[12]; Le et al., 2013[9]; Cheever et al., 2009[1]). Such approaches might be of particular interest for triple-negative breast cancers, a subtype where currently few treatment options are available in addition to chemotherapy, and that has been reported to have higher levels of TILs compared with other subtypes (Vonderheide et al., 2017[19]; Katz and Alsharedi, 2017[8]).

In this field, Lina Liu and colleagues recently published an interesting study in which they induced a cytotoxic T-lymphocyte response in a mouse model of triple negative breast cancer (Liu et al., 2018[11]). The authors used nanoparticles as carriers to deliver mRNA encoding the transmembrane protein MUC1 to dendritic cells in lymph nodes. Carcinomas of the breast, ovary, colon, rectum, pancreas and prostate are known to overexpress MUC1. Administration of MUC1 mRNA loaded nanoparticles clearly reduced tumor growth in the mice. Combined administration of MUC1 mRNA plus an anti-CTLA-4 antibody additionally reduced tumor size compared to treatment with MUC1 mRNA alone. Liu and colleagues are to be congratulated for their interesting results. Future research will show whether the MVL1 mRNA nano-vaccine will be successful in translational studies. 

## References

[1] Cheever MA, Allison JP, Ferris AS, Finn OJ, Hastings BM, Hecht TT (2009). The priorization of cancer antigens: a national cancer institute pilot project for the acceleration of translational research. Clin Cancer Res.

[2] Chen Z, Gerhold-Ay A, Gebhard S, Boehm D, Solbach C, Lebrecht A (2012). Immunoglobulin kappa C predicts overall survival in node-negative breast cancer. PLoS One.

[3] Choi SY, Collins CC, Gout PW, Wang Y (2013). Cancer-generated lactic acid: a regulatory, immunosuppressive metabolite?. J Pathol.

[4] Godoy P, Cadenas C, Hellwig B, Marchan R, Stewart J, Reif R (2014). Interferon-inducible guanylate binding protein (GBP2) is associated with better prognosis in breast cancer and indicates an efficient T cell response. Breast Cancer.

[5] Heimes AS, Madjar K, Edlund K, Battista MJ, Almstedt K, Elger T (2017). Subtype-specific prognostic impact of different immune signatures in node-negative breast cancer. Breast Cancer Res Treat.

[6] Heimes AS, Madjar K, Edlund K, Battista MJ, Almstedt K, Gebhard S (2017). Prognostic significance of interferon regulating factor 4 (IRF4) in node-negative breast cancer. J Cancer Res Clin Oncol.

[7] Jiang T, Xu X, Qiao M, Li X, Zhao C, Zhou F (2018). Comprehensive evaluation of NT5E/CD73 expression and its prognostic significance in distinct types of cancers. BMC Cancer.

[8] Katz H, Alsharedi M (2017). Immunotherapy in triple-negative breast cancer. Med Oncol.

[9] Le DT, Lutz E, Uram JN, Sugar EA, Onners B, Solt S (2013). Evaluation of ipilimumab in combination with allogeneic pancreatic tumor cells transfected with a GM-CSF gene in previously treated pancreatic cancer. J Immunother.

[10] Leone RD, Emens LA (2018). Targeting adenosine for cancer immunotherapy. J Immunother Cancer.

[11] Liu L, Wang Y, Miao L, Liu Q, Musetti S, Li J (2018). Combination immunotherapy of MUC1 mRNA nano-vaccine and CTLA-4 blockade effectively inhibits growth of triple negative breast cancer. Mol Ther.

[12] Melero I, Gaudernack G, Gerritsen W, Huber C, Parmiani G, Scholl S (2014). Therapeutic vaccines for cancer: an overview of clinical trials. Nat Rev Clin Oncol.

[13] Pennock GK, Chow LQ (2015). The evolving role of immune checkpoint inhibitors in cancer treatment. Oncologist.

[14] Schmidt M, Böhm D, von Törne C, Steiner E, Puhl A, Pilch H (2008). The humoral immune system has a key prognostic impact in node-negative breast cancer. Cancer Res.

[15] Schmidt M, Hellwig B, Hammad S, Othman A, Lohr M, Chen Z (2012). A comprehensive analysis of human gene expression profiles identifies stromal immunoglobulin κ C as a compatible prognostic marker in human solid tumors. Clin Cancer Res.

[16] Schmidt M, Weyer-Elberich V, Hengstler JG, Heimes AS, Almstedt K, Gerhold-Ay A (2018). Prognostic impact of CD4-positive T cell subsets in early breast cancer: a study based on the FinHer trial patient population. Breast Cancer Res.

[17] Sharma P, Allison JP (2015). The future of immune checkpoint therapy. Science.

[18] Sota Y, Naoi Y, Tsunashima R, Kagara N, Shimazu K, Maruyama N (2014). Construction of novel immune-related signature for prediction of pathological complete response to neoadjuvant chemotherapy in human breast cancer. Ann Oncol.

[19] Vonderheide RH, Domchek SM, Clark AS (2017). Immunotherapy for breast cancer. What are we missing?. Clin Cancer Res.

[20] Xu Z, Ramishetti S, Tseng YC, Guo S, Wang Y, Huang L (2013). Multifunctional nanoparticles co-delivering Trp2 peptide and CpG adjuvant induce potent cytotoxic T-lymphocyte response against melanoma and its lung metastasis. J Control Release.

